# Inflammation in Carpal Tunnel Syndrome: A Systematic Review and Meta-Analysis of the Immune Profile

**DOI:** 10.3390/jcm15145545

**Published:** 2026-07-15

**Authors:** Ireneusz Walaszek, Kaja Giżewska-Kacprzak, Szymon Grochans, Kamila Rachubińska, Elżbieta Grochans, Karolina Skonieczna-Żydecka, Anna Maria Cybulska

**Affiliations:** 1 Department of Nursing, Faculty of Health Sciences, Pomeranian Medical University in Szczecin, 48 Żołnierska St., 71-210 Szczecin, Poland; ireneusz.walaszek@pum.edu.pl (I.W.); kamila.rachubinska@pum.edu.pl (K.R.); elzbieta.grochans@pum.edu.pl (E.G.); anna.cybulska@pum.edu.pl (A.M.C.); 2 Department of Pediatric and Oncological Surgery, Urology and Hand Surgery, Pomeranian Medical University in Szczecin, 1 Unii Lubelskiej Street, 71-252 Szczecin, Poland; kaja.gizewska.kacprzak@pum.edu.pl (K.G.-K.); szymon.grochans@pum.edu.pl (S.G.); 3 Department of Biochemical Science, Pomeranian Medical University in Szczecin, 71-460 Szczecin, Poland

**Keywords:** carpal tunnel syndrome, chronic inflammation, cytokines, inflammatory markers, pathophysiology

## Abstract

**Objectives:** This systematic review and meta-analysis aimed to assess whether serum inflammatory markers are associated with Carpal Tunnel Syndrome (CTS), moving beyond the traditional mechanical explanation. **Methods:** Seven observational studies involving a total of 609 adult participants (CTS patients and matched healthy controls) were included. Studies were selected through comprehensive searches of PubMed and Embase databases up to April 2025. Data extraction and quality assessment were independently performed by two reviewers using the Newcastle–Ottawa Scale. Standardized mean differences (SMDs) and 95% confidence intervals were calculated using random-effects meta-analysis. **Results:** CTS patients exhibited significantly elevated serum levels of CCL2, CCL5, CXCL10, CXCL8, IL-4, VEGF, and TGF-β compared to controls. CCL4 was increased in the fixed-effect model but showed high heterogeneity; however, it was not statistically significant in the random-effects model and demonstrated extreme heterogeneity. No significant or consistent differences were observed for IL-1β, IL-6, IL-9, IL-10, TNF-α, or IFN-α. **Conclusions:** CTS is associated with a systemic pro-inflammatory and pro-fibrotic profile. CCL2, CCL5, IL-4, VEGF, and TGF-β may serve as promising biomarkers. Further large-scale, standardized studies are needed to validate these findings.

## 1. Introduction

### 1.1. Carpal Tunnel Syndrome

Carpal tunnel syndrome (CTS) is the most common compression neuropathy of the median nerve and the most prevalent entrapment neuropathy of the upper extremity, affecting approximately 3% of the general population. It occurs more frequently in women, with a mean age at diagnosis of around 50 years [1]. CTS is typically characterized by symptoms localized to the hand, particularly the thumb, index, and middle fingers, as well as the radial half of the ring finger, corresponding to the sensory distribution of the median nerve. In more severe cases, symptoms may radiate proximally to the forearm, upper arm, or even the shoulder. When such proximal spread occurs, the symptoms are more often painful rather than paresthetic, and may be misinterpreted as originating from other conditions, such as cervical radiculopathy or shoulder pathology.

Sensory disturbances are the most commonly reported features of CTS, with numbness being the predominant symptom rather than pain. Symptoms typically worsen at night, and patients frequently report intermittent nocturnal paresthesias and dysesthesias. Over time, these symptoms may increase in frequency and also occur during daytime activities, particularly those involving sustained wrist flexion or repetitive hand movements [2,3,4]. In long-standing cases, involvement of motor fibers of the median nerve may occur, leading to clinically evident signs such as thenar muscle atrophy. These findings are indicative of advanced nerve compression and are usually readily recognized by clinicians. Prompt intervention is essential, as motor deficits may become irreversible if treatment is delayed [5]. CTS may also involve disruption of axoplasmic flow within the autonomic fibers of the median nerve, potentially resulting in autonomic dysfunction. These manifestations are often under-recognized by both patients and clinicians. They may include alterations in local blood flow, thermoregulation, sweating, and trophic changes within the median nerve sensory territory. Impairment of sympathetic axonal transport can lead to reduced sympathetic activity and subsequent vasodilation. Clinically, the affected area may appear swollen, warmer, more erythematous, and drier compared to the surrounding tissue. Changes in skin moisture may contribute to hyperkeratosis, eczema, xerosis, and delayed wound healing. In rare and severe cases, compromised vascular supply may lead to fingertip ulceration and nail growth abnormalities. Additionally, Raynaud’s phenomenon has been reported in 10% to 60% of patients with CTS. In advanced stages, autonomic dysfunction may result in irreversible structural changes such as osteoporosis and acro-osteolysis [3].

Several risk factors have been associated with CTS, including diabetes mellitus, menopause, hypothyroidism, obesity, arthritis, and pregnancy. Although hormonal factors have been proposed as potential contributors, current evidence does not definitively support this hypothesis [3,6,7]. CTS remains primarily a clinical diagnosis, based on a constellation of characteristic symptoms, including nocturnal paresthesias, hand numbness, and weakness in the median nerve distribution. However, anatomical variations may complicate clinical presentation, making diagnosis more challenging in certain cases [8].

### 1.2. CTS and Inflammatory Cytokines: A Revealed Pathophysiological Connection

CTS is primarily considered an idiopathic condition; however, it has also been associated with various metabolic and inflammatory diseases [9,10]. Recent studies increasingly suggest that inflammatory mediators in the bloodstream may play a role during the active phase of CTS [11].

Current evidence indicates that CTS may be driven by sterile inflammation and an imbalance in oxidative stress. Sterile inflammation refers to an inflammatory response not caused by infection, but rather triggered by tissue damage, overuse, or biomechanical stress [12]. In CTS, prolonged compression within the carpal tunnel initiates a local inflammatory response, leading to the release of pro-inflammatory cytokines (such as IL-6 and TNF-α) and cellular stress mediators. This process contributes to swelling and thickening of the flexor tendon sheaths, which further increase pressure within the carpal tunnel and exacerbates symptoms [13].

At the same time, oxidative stress arises from an imbalance between the production of reactive oxygen species (ROS) and the body’s capacity to neutralize them. ROS can damage cellular components, including proteins, lipids, and DNA, thereby contributing to median nerve degeneration and the persistence of neurological symptoms. Additionally, ROS may enhance the expression of inflammatory cytokines, creating a self-perpetuating cycle of inflammation and tissue injury [14].

The interaction between sterile inflammation and oxidative stress may explain why CTS can progress even in the absence of clear anatomical abnormalities and why symptoms may persist following surgical decompression [15]. Moreover, several studies have demonstrated that elevated levels of inflammatory and oxidative stress markers are significantly associated with greater clinical severity of CTS [16].

Emerging evidence also supports the presence of systemic inflammation in CTS, suggesting that entrapment neuropathy may involve alterations in memory T-cell homeostasis and increased levels of circulating cytokines and chemokines, which may contribute to neuropathic symptoms [17]. Also, a Mendelian randomization approach suggested a potential causal link between selected inflammatory cytokines and CTS. However, such findings should be interpreted with caution, as they rely on genetic instrumental variables and do not replace evidence from longitudinal or interventional studies [18]. Also, inconsistent findings across studies challenge this association [19], underscoring the need for further research on inflammatory biomarkers in CTS.

The aim of this systematic review and meta-analysis was to evaluate the association between inflammatory marker levels and carpal tunnel syndrome based on the available evidence. Specifically, we investigated whether inflammatory cytokines are altered in patients with CTS and assessed their potential role as biomarkers of disease pathophysiology. We hypothesized that CTS is associated with increased levels of selected pro-inflammatory cytokines compared with healthy controls.

## 2. Materials and Methods

### 2.1. Search Strategy and Selection Criteria

This study was conducted according to the requirements included in the Preferred Reporting Items for Systematic Reviews and Meta-Analyses (PRISMA) protocols. The completed PRISMA 2020 Checklist is provided in the Supplementary Materials. Two independent authors systematically searched PubMed/Embase from database inception until 17 April 2025.

The following search terms with medical subject headings for PUBMED were used: ((“CTS” [All Fields] OR “CTS” [MeSH Terms] AND (“cytokine” [All Fields] OR “cytokine” [MeSH Terms]) OR “chemokine” [All Fields] OR “chemokine” [MeSH Terms])), and for EMBASE (‘CTS’/exp AND (‘cytokine’/exp OR ‘cytokine’ OR ‘cytokines’ OR ‘interleukin’ OR ‘chemokine’/exp).

The electronic search was supplemented by a manual review of the reference lists from eligible publications and relevant reviews.

Inclusion criteria for studies were as follows:

- Human studies (either observational or clinical; for clinical studies, only baseline data were considered);
- Confirmed diagnosis of CTS;
- Availability of data on serum levels of chemokines, interleukins, or other inflammatory markers measured prior to any treatment.

Exclusion criteria for studies were as follows:

- Studies in animals;
- Diagnosis of primary hand trauma;
- Studies in children or pregnant women;
- No control group.

This systematic review was conducted in accordance with the PRISMA (Preferred Reporting Items for Systematic Reviews and Meta-Analyses) guidelines. The protocol of the present study has been registered in the PROSPERO database (CRD:1103887). The exclusion of studies without a control group was a deliberate methodological decision, aligned with the objective of this meta-analysis, which was to quantitatively compare levels of inflammatory markers between patients with carpal tunnel syndrome (CTS) and healthy individuals. The absence of a control group prevents the comparison of biomarker levels against an appropriate reference population, significantly limiting the interpretability of the findings and precluding their inclusion in comparative analyses. Furthermore, including such studies could increase heterogeneity and reduce the reliability of the estimated effect size, thereby compromising the overall consistency and robustness of the analysis.

### 2.2. Data Extraction and Analysis

Two authors (K.G-K. and G. Sz.) independently extracted data from each study, including details on study characteristics (e.g., study design, focus, sponsorship), participant demographics (e.g., age and sex), and CTS-related information (e.g., disease duration, severity), as well as serum levels of inflammatory markers such as chemokines and interleukins. A third author (A.M.C.) independently verified the extracted data to ensure accuracy and consistency. When data were presented only in graphical form, values were extracted using WebPlotDigitizer software version 5 (https://automeris.io/WebPlotDigitizer/ (accessed on 15 December 2025).

### 2.3. Outcomes

The primary outcome was data on the concentrations of any inflammatory marker in CTS compared to healthy controls.

### 2.4. Data Synthesis and Statistical Analysis

We conducted a random-effects meta-analysis of outcomes for which ≥2 studies contributed data using Comprehensive Meta-Analysis V3 (http://www.meta-analysis.com, accessed on 15 December 2025). The effect size was expressed as standardized mean difference (SMD). We explored study heterogeneity using the chi-square test of homogeneity, with *p* < 0.05 indicating significant heterogeneity. All analyses were two-tailed with an alpha = 0.05.

### 2.5. Risk of Bias Assessment

Studies were evaluated using the NOS, which measures study quality across three domains: Selection (max 4 points), Comparability (max 2 points), and Exposure/Outcome (max 3 points).

- Selection: Scores ranged from 2 to 4, with most studies achieving the maximum score, indicating generally appropriate case selection and representativeness.
- Comparability: Scores varied from 0 to 2, showing differences in how well studies controlled for confounding variables; one study had no adjustment for comparators.
- Exposure/Outcome: Scores ranged from 2 to 3, suggesting that most studies adequately measured outcomes, though some had minor limitations.

Total scores ranged from 4 to 9 (max 9):

- High quality: four studies (scores 8–9)
- Good quality: one study (score 7)
- Moderate quality: one study (score 6)
- Low quality: one study (score 4)

Two reviewers (S.Z.G. and K.R.) independently assessed the risk of bias in the included studies using the Newcastle–Ottawa Scale (NOS). Any discrepancies were resolved through consultation with a third reviewer (A.M.C). Studies were classified as high quality if they received a score of at least 7 points (corresponding to 7 stars) according to the NOS criteria.

## 3. Results

### 3.1. Search Results

**Figure 1:**
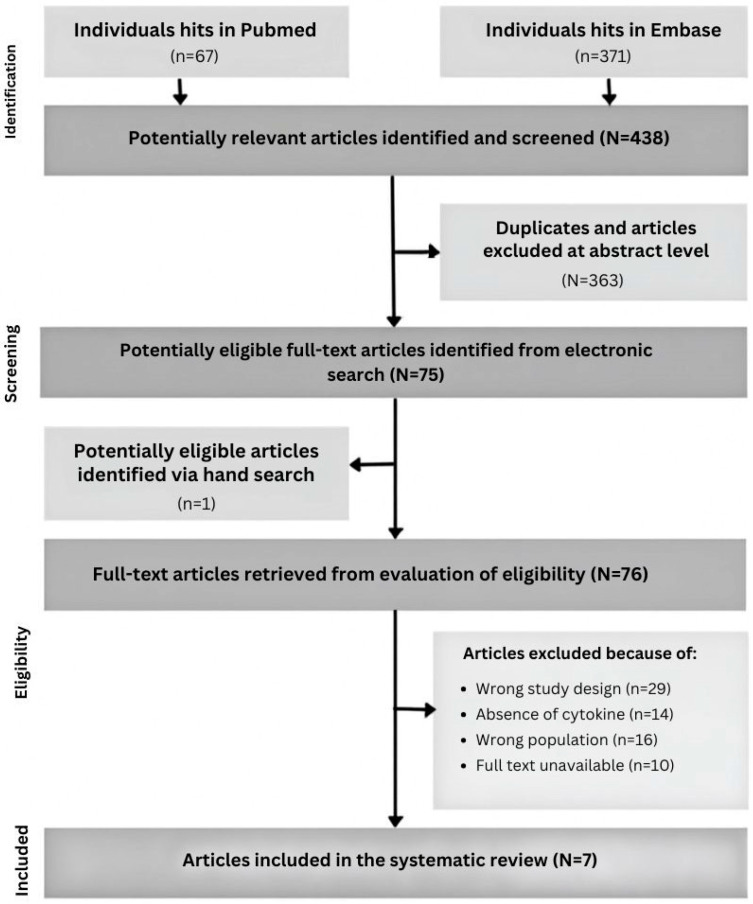
Flow chart.

The literature search identified 438 records, of which 76 full-text articles were assessed for eligibility after screening. Ultimately, seven studies met the inclusion criteria and were included in the meta-analysis (Figure 1).

### 3.2. Characteristics of the Included Studies

This meta-analysis included seven studies published between 2017 and 2023 [11,13,17,19,20,21,22] and comprised a total of 609 participants, predominantly female (74.7%). Sample sizes ranged from 52 to 200 individuals. Studies were conducted across multiple regions, including Europe, Asia, Australia, Egypt, and the United States. All studies were observational and included a control group. Detailed characteristics are presented in Table 1.

The majority of patients presented with bilateral CTS (n = 107). In 72 respondents, symptoms were limited to the right hand, while in 51 they affected only the left hand. Three studies did not provide information on the localization of CTS symptoms. Details are presented in Table 1.

In the included studies, the diagnosis of carpal tunnel syndrome was generally based on a combination of clinical assessment and electrodiagnostic testing, although the specific approaches varied slightly between studies. Most authors incorporated characteristic clinical symptoms (such as hand pain, paresthesia, and numbness in the median nerve distribution) along with physical examination findings, including positive provocative tests (e.g., Tinel’s sign and Phalen’s maneuver) and signs of thenar muscle involvement. Several studies [11,21] applied comprehensive diagnostic criteria that combined patient history, clinical signs, and nerve conduction abnormalities, in some cases following established guidelines (e.g., American Academy of Neurology criteria). In addition, many studies [13,19,20,22] confirmed the diagnosis using electrodiagnostic methods such as electromyography and nerve conduction studies, which were often considered essential for objective verification. Overall, the diagnostic approach across studies was relatively consistent, relying on both clinical presentation and electrophysiological confirmation to ensure accurate identification of CTS cases.

In most of the included studies [11,13,19,20,21,22], exclusion criteria were consistently implemented to minimize potential confounding factors and reduce the likelihood of including conditions that could mimic or influence the presentation of carpal tunnel syndrome. These criteria typically included systemic diseases (such as diabetes mellitus, thyroid disorders, kidney disease, amyloidosis, gout, and malignancies), as well as inflammatory and autoimmune conditions (including rheumatoid arthritis, systemic lupus erythematosus, and fibromyalgia). Furthermore, participants with neurological disorders (e.g., neuropathies or radiculopathies), a history of upper limb trauma or surgery, and other musculoskeletal conditions affecting the upper extremity were commonly excluded. Additional exclusions often comprised pregnancy, active infections, use of corticosteroids or immunosuppressive therapy, smoking, obesity, and other metabolic disorders.

### 3.3. The Risk of Bias Assessment

Overall, most studies were of high methodological quality according to the Newcastle–Ottawa Scale, although variability in the control of confounding factors and exposure assessment was noted. NOS domain scores indicated generally appropriate case selection (2–4 points), variable control of confounding factors (0–2 points), and mostly adequate exposure/outcome assessment (2–3 points), with some minor methodological limitations. Total NOS scores ranged from 4 to 9, with most studies classified as high quality (n = 4), and the remainder as good (n = 1), moderate (n = 1), or low quality (n = 1).

### 3.4. Meta-Analysis

#### Outcome Measures

Serum Levels of Chemokines in Patients With and Without CTS

**Figure 2:**
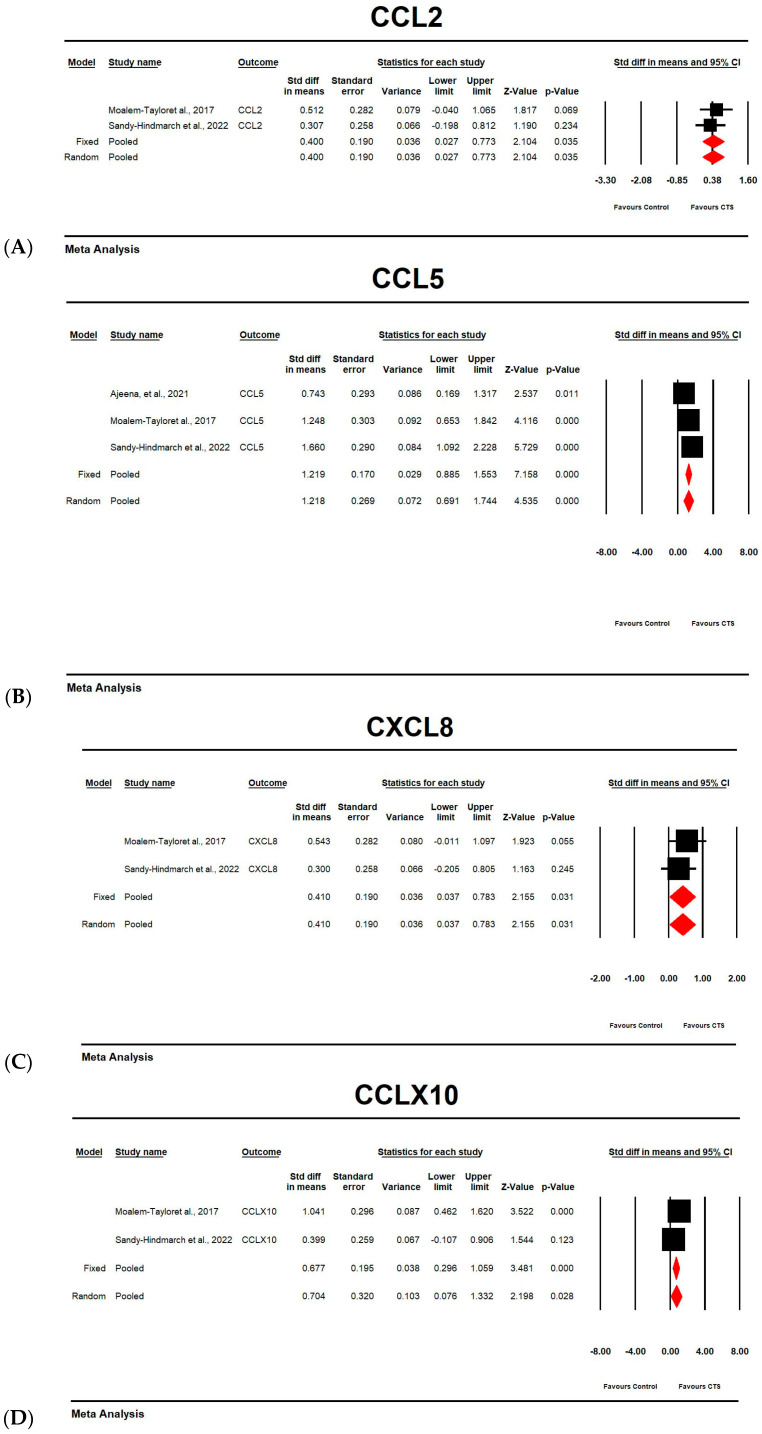
Effect size for CCL2 levels in patients with CTS compared to individuals without the diagnosis: Q = 0.289, df(Q) = 1, *p* = 0.591, I^2^ = 0.000 [13,17] (**A**). Effect size for CCL5 levels in patients with CTS compared to individuals without the diagnosis: Q = 4.965, df(Q) = 2, *p* = 0.084, I^2^ = 59.717 [13,17,20] (**B**). Effect size for CXCL8 levels in patients with CTS compared to individuals without the diagnosis: Q = 0.405, df(Q) = 1, *p* = 0.524, I^2^ = 0.000 [13,17] (**C**). Effect size for CXCL10 levels in patients with CTS compared to individuals without the diagnosis: Q = 2.669, df(Q) = 1, *p* = 0.102, I^2^ = 62.532 (**D**) [13,17]. Black squares represent the effect size of individual studies, while red diamonds indicate the combined effect sizes calculated using mixed and random-effects models.

Significantly higher serum levels of several chemokines were observed in CTS patients compared to controls. Elevated concentrations were found for (Figure 2):

- CCL2 (SMD = 0.400; 95% CI: 0.027–0.773; *p* = 0.035; I^2^ = 0%);
- CCL5 (SMD = 1.218; 95% CI: 0.691–1.744; *p* < 0.001; I^2^ = 59.7%);
- CXCL8 (SMD = 0.410; 95% CI: 0.037–0.783; *p* = 0.031; I^2^ = 0%);
- CXCL10 (SMD = 0.704; 95% CI: 0.076–1.332; *p* = 0.028; I^2^ = 62.5%).

**Figure 3:**
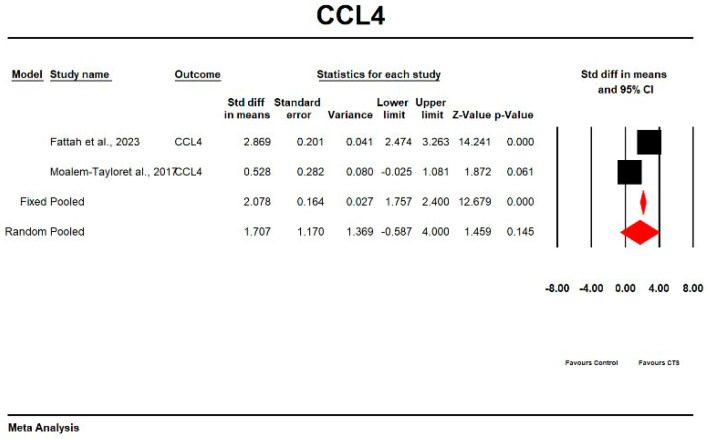
Effect size for CCL4 levels in patients with CTS compared to individuals without the diagnosis: Q = 45.578, df (Q) = 1, *p* = 0.000, I^2^ = 97.806 [17,21]. Black squares represent the effect size of individual studies, while red diamonds indicate the combined effect sizes calculated using mixed and random-effects models.

For CCL4, results were inconsistent, with no statistically significant effect in the random-effects model (SMD = 1.707; 95% CI: −0.587 to 4.000; *p* = 0.145) and substantial heterogeneity (I^2^ = 97.8%). The results hereby suggest that elevated chemokine levels are associated with CTS and may reflect underlying inflammatory processes. Detailed data is presented in Figure 3.

### 3.5. Serum Levels of Interleukins in Patients with and Without CTS

Among interleukins, only IL-4 showed significantly higher levels in CTS patients (SMD = 0.696; 95% CI: 0.203–1.189; *p* = 0.006; I^2^ = 40.6%).

**Figure 4:**
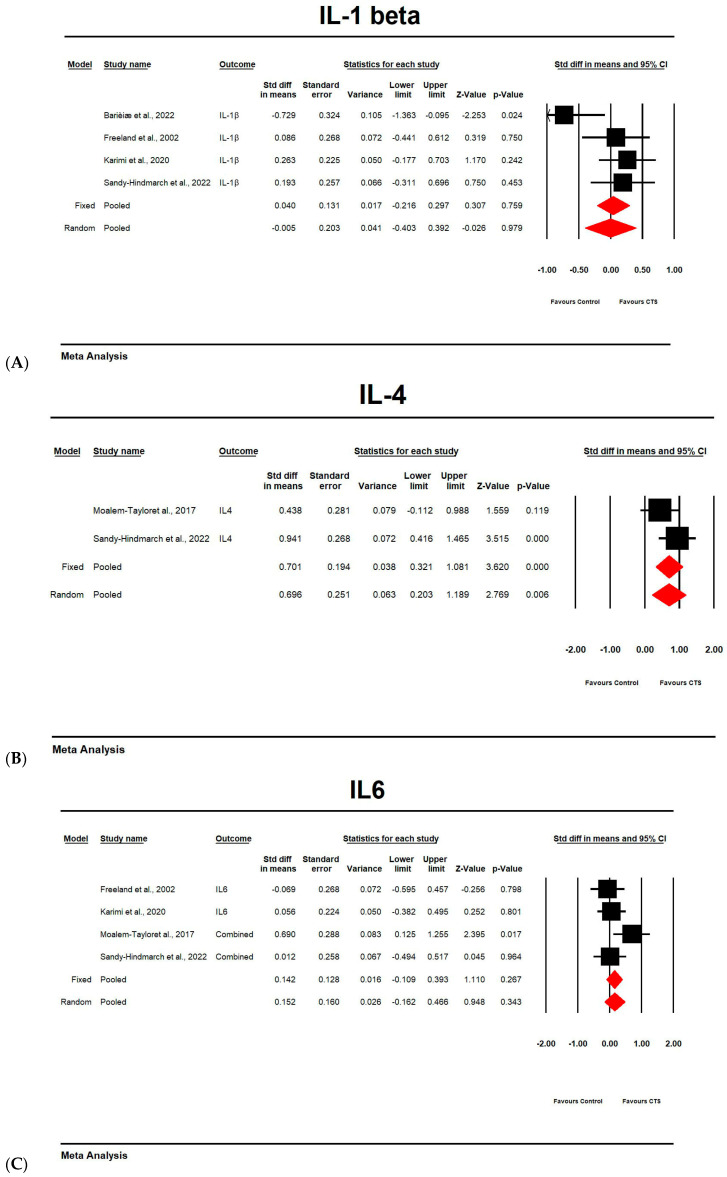

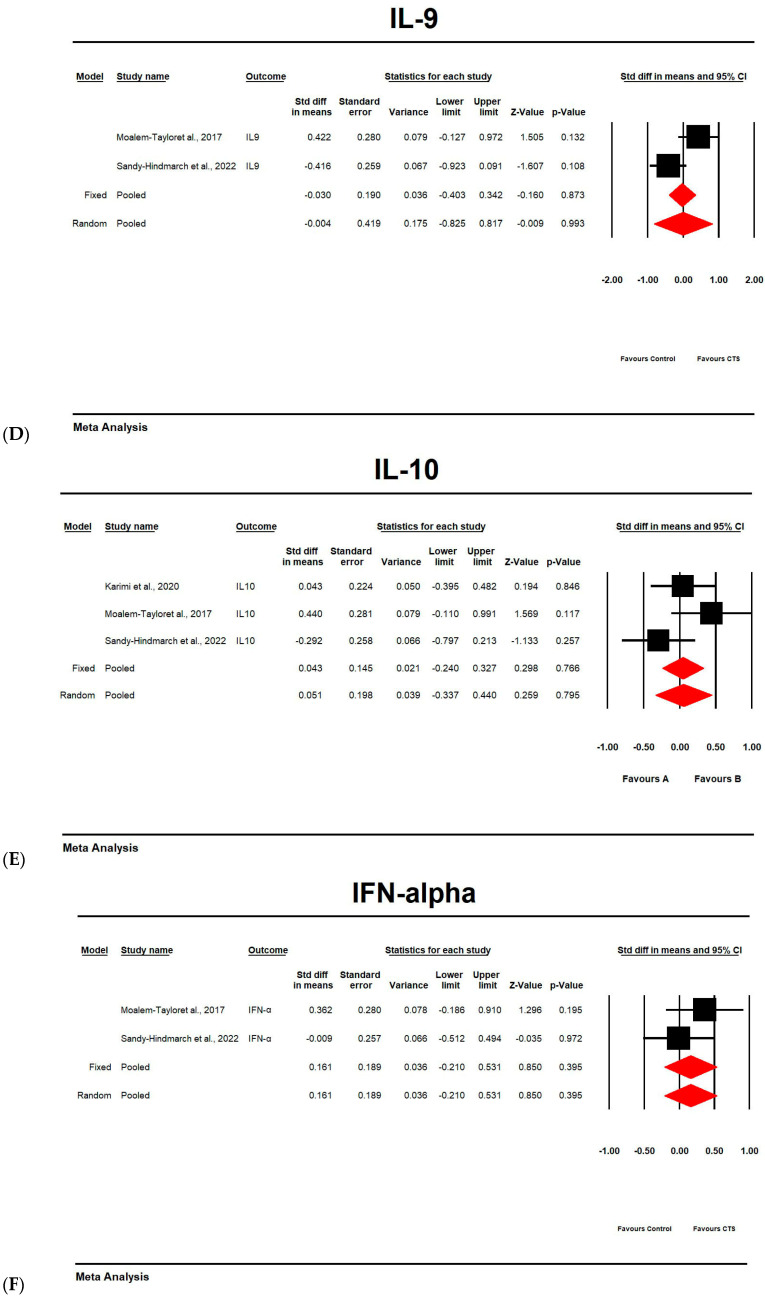
Effect size for IL-1β levels in patients with CTS compared to individuals without the diagnosis: Q = 7.015, df(Q) = 3, *p* = 0.071, I^2^ = 57.237 [11,13,19,22] (**A**). Effect size for IL-4 levels in patients with CTS compared to individuals without the diagnosis: Q = 1.685, df(Q) = 1, *p* = 0.195, I^2^ = 40.553 [13,17] (**B**). Effect size for IL-6 levels in patients with CTS compared to individuals without the diagnosis: Q = 4.638, df(Q)= 3, *p* = 0.200, I^2^ = 35,316 [13,17,19,22] (**C**). Effect size for IL-9 levels in patients with CTS compared to individuals without the diagnosis: Q = 4.822, df(Q)= 1, *p* = 0.028, I^2^ = 79.262 [13,17] (**D**). Effect size for IL-10 levels in patients with CTS compared to individuals without the diagnosis: Q = 3.694, df(Q) = 2, *p* = 0.158, I^2^ = 45.864 [13,17,19] (**E**). Effect size for IFN-α levels in patients with CTS compared to individuals without the diagnosis: Q = 0.958, df(Q) = 1, *p* = 0.328, I^2^ = 0.000 [13,17] (**F**). Black squares represent the effect size of individual studies, while red diamonds indicate the combined effect sizes calculated using mixed and random-effects models.

No statistically significant differences were observed for (Figure 4):

- IL-1β (SMD = −0.005; 95% CI: −0.403 to 0.392; *p* = 0.979; I^2^ = 57.2%);
- IL-4 (SMD = 0.696; 95% CI: 0.203 to 1.189; *p* = 0.006; I^2^ = 40.6%);
- IL-6 (SMD = 0.142; 95% CI: −0.109 to 0.393; *p* = 0.267; I^2^ = 35.3%);
- IL-9 (SMD = −0.004; 95% CI: −0.825 to 0.817; *p* = 0.993 I^2^ = 79.3%);
- IL-10 (SMD = 0.051; 95% CI: −0.337 to 0.259; *p* = 0.795 I^2^ = 45.9%);
- IFN-α (SMD = 0.161; 95% CI: −0.210 to 0.531; *p* = 0.395 I^2^ = 0%).

### 3.6. Other Inflammatory Markers in Patients with and Without CTS

**Figure 5:**
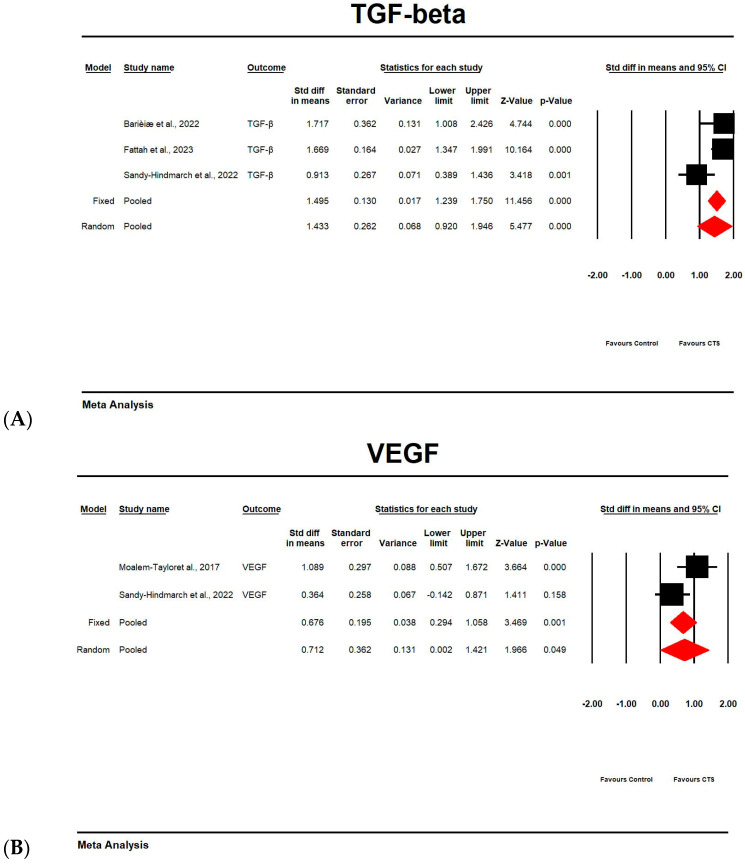

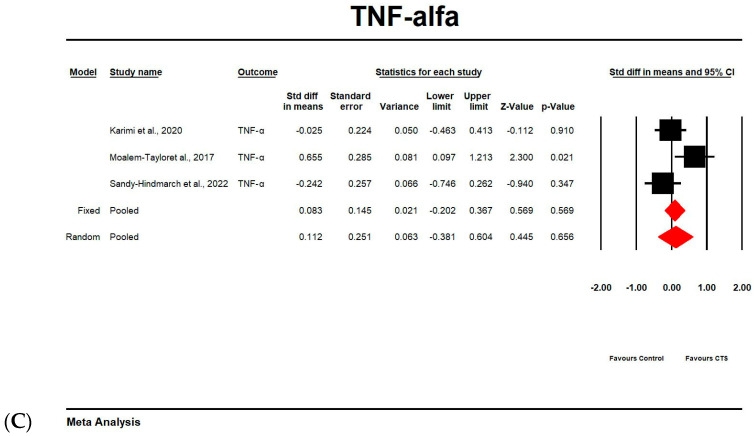
Effect the size for TGF-β levels in patients with CTS compared to individuals without the diagnosis: Q = 6.257, df(Q)= 2, *p* = 0.044, I^2^ = 68.034 [11,13,21] (**A**). Effect size for VEGF levels in patients with CTS compared to individuals without the diagnosis: Q = 3.388, df(Q)= 1, *p* = 0.066, I^2^ = 70.483 [13,17] (**B**). Effect size for TNF-α levels in patients with CTS compared to individuals without the diagnosis: Q = 5.861, df(Q)= 2, *p* = 0.053, I^2^ = 68.879 [13,17,19] (**C**). Black squares represent the effect size of individual studies, while red diamonds indicate the combined effect sizes calculated using mixed and random-effects models.

Significantly elevated serum levels were also observed for (Figure 5):

- TGF-β (SMD = 1.433; 95% CI: 0.920–1.946; *p* < 0.001; I^2^ = 68.0%)
- VEGF (SMD = 0.712; 95% CI: 0.002–1.421; *p* = 0.049; I^2^ = 70.5%).

In contrast, TNF-α showed no significant association with CTS (SMD = 0.112; 95% CI: −0.381 to 0.604; *p* = 0.656 I^2^ = 68.9%) (Figure 5).

### 3.7. Publication Bias

Funnel plots were visually inspected and did not reveal significant asymmetry. Egger’s regression test did not indicate evidence of publication bias for the analyzed outcomes (all *p* > 0.05), although the small number of studies for some markers limits the reliability of these assessments.

## 4. Discussion

In this literature review, the available scientific evidence on the associations between inflammatory markers—particularly chemokines, interleukins, and other cytokines—and carpal tunnel syndrome was analyzed. The results of the conducted analysis indicate significant associations between CTS and selected inflammatory markers, including chemokines, interleukins, and growth factors. The findings suggest that, although CTS is classically regarded as a neuropathy resulting from mechanical compression of the median nerve, its pathogenesis may involve inflammatory and immunological processes. The detection of elevated levels of certain inflammatory mediators in CTS patients suggests the possible involvement of a systemic inflammatory response in the pathogenesis and clinical course of the disease [11,13,17,19,20,21,22]. An important aspect supporting the involvement of inflammatory mechanisms is the marked female predominance in CTS. Women are affected approximately two to three times more frequently than men, suggesting that sex-related biological factors influence disease susceptibility. Hormonal factors, particularly fluctuations in estrogen levels, may alter connective tissue metabolism, vascular permeability, and immune responses, while genetic susceptibility and sex-specific immune regulation may further contribute. These mechanisms could partly explain the higher incidence of CTS in women, although their precise role remains to be established [8,23,24]. Another important consideration is the interaction between mechanical loading and inflammation. Mechanical compression of the median nerve likely initiates a cascade of mechanobiological events rather than acting in isolation. Compression, repetitive strain, and increased intracarpal pressure may activate fibroblasts, Schwann cells, endothelial cells, and resident immune cells through mechanotransduction pathways, promoting inflammatory mediator release, extracellular matrix remodeling, fibrosis, and edema. These changes may further increase carpal tunnel pressure, creating a self-perpetuating cycle of mechanical stress and inflammation. Thus, mechanobiological factors should be considered integral to CTS pathogenesis [10]. Overall, CTS appears to be a multifactorial disorder in which mechanical compression, mechanobiological signaling, and inflammatory processes interact to drive disease onset and progression. Future prospective studies integrating cytokine profiling with histopathological, biomechanical, and molecular analyses are needed to clarify these interactions and identify potential therapeutic targets beyond surgical decompression [2,3,4,5].

The meta-analysis revealed a heterogeneous pattern of associations between inflammatory mediators and CTS. Significant elevations in serum levels were consistently observed for several chemokines and cytokines, including CCL2, CCL5, CXCL10, CXCL8, IL-4, TGF-β, and VEGF, suggesting their potential involvement in CTS pathophysiology. These markers demonstrated moderate–large effect sizes; however, given the observational nature of the included studies, these findings should be interpreted as associative rather than causal. CCL4 also showed a large difference in fixed-effect analysis, but high between-study variability limited the robustness of this association [11,13,17,19,20,21,22].

Conversely, IL-1β, IL-6, IL-9, IL-10, TNF-α, and IFN-α did not show consistent or statistically significant differences between CTS patients and controls in pooled analyses, despite some isolated significant findings at the single-study level. These null results suggest that their role, if any, may be limited, context-dependent, or masked by methodological heterogeneity [11,13,17,19,20,21,22].

Overall, the evidence points toward a pro-inflammatory and possibly pro-fibrotic serum profile in CTS, with certain chemokines (CCL2, CCL5, CXCL10) and growth factors (VEGF, TGF-β) emerging as the most promising biomarkers. These findings warrant further investigation in larger, standardized studies to clarify causal relationships, explore temporal dynamics, and assess their potential as diagnostic or prognostic markers [11,13,17,19,20,21,22].

### 4.1. Serum Levels of Chemokines in Patients With and Without CTS

These chemokines are key mediators of the inflammatory process, responsible for recruiting monocytes, T lymphocytes, and other immune cells to the site of nerve injury [25]. The meta-analysis revealed significantly higher serum concentrations of CCL2, CCL5, CXCL10, and CXCL8 in patients diagnosed with carpal tunnel syndrome (CTS) compared with control groups. These findings indicate activation of multiple immune pathways in the course of CTS, suggesting a complex inflammatory mechanism underlying the condition. CCL2 levels were higher in individuals with CTS compared to those without the diagnosis. Both CCL2 (MCP-1) and CCL5 (RANTES) are critical chemokines involved in leukocyte recruitment and play an important role in inflammatory response and peripheral nerve regeneration [26,27,28].

Elevated levels of CXCL8 (IL-8)—a potent neutrophil chemoattractant—may indicate an active inflammatory response within the median nerve and surrounding structures. CXCL8 and CXCL10 concentrations were higher in individuals with CTS compared with other respondents [13,17]. The presence of higher CXCL10 levels, which is less frequently described in the context of CTS, suggests the potential involvement of alternative chemotactic pathways requiring further investigation. The absence of significant differences for CCL4 may result from high heterogeneity of the analyzed studies and methodological variations [8,13,17,28].

### 4.2. Serum Levels of Interleukins in Patients With and Without CTS

Among the interleukins analyzed, only IL-4 showed significantly higher concentrations in CTS patients, which may suggest a shift in the immune response toward a Th2 profile. IL-4 is associated with humoral immunity and repair processes; however, its role in tissue remodeling and fibrosis may contribute to flexor retinaculum thickening and the development of CTS [13,17].

For classical pro-inflammatory cytokines such as IL-1β, IL-6, IL-9, and IL-10, no significant differences were observed between groups. This may reflect the local (rather than systemic) nature of the inflammatory response, temporal variability in cytokine expression, or confounding factors such as comorbidities or pharmacological treatment [11,13,17,19,22].

The lack of consistent results for interleukin 6 (IL-6)—one of the most frequently studied cytokines in the context of entrapment neuropathies—is particularly noteworthy. In some studies, such as that by Miyasaka et al. [27], local overproduction of IL-6 in the tendon sheath tissue was demonstrated in patients with dialysis-related amyloidosis and concurrent CTS. Ajeena et al. [20] found non-significant differences in IL-6 levels, challenging earlier reports of its key role in CTS. Similarly, Karimi et al. [19] did not confirm elevated IL-6 serum levels in idiopathic CTS patients. Such discrepancies may stem from population heterogeneity, temporal variability in cytokine expression, and the presence of confounding factors such as comorbidities (e.g., diabetes, obesity) or concurrent medication use. Shareef et al. [29] noted considerable variation in IL-6 findings and emphasized the need for larger, well-designed studies controlling for confounders.

### 4.3. Other Inflammatory Markers in Patients With and Without CTS

Significantly higher serum levels of TGF-β and VEGF were observed in CTS patients. TGF-β, known for its pro-fibrotic properties, may contribute to synovial membrane fibrosis and thickening of the carpal tunnel structures. Persistently elevated TGF-β may also limit nerve regeneration after surgical decompression. Elevated VEGF levels may reflect microcirculatory changes and an angiogenic response to local hypoxia within the carpal tunnel [13,17,21].

The findings reported by Gingery et al. further support the growing evidence that fibrosis is an important component of CTS pathophysiology. They demonstrated that fibrosis of the subsynovial connective tissue (SSCT) is characterized by increased expression of pro-fibrotic mediators, including TGF-β, CTGF, collagen I, collagen III, and SMAD3, which may contribute to progressive median nerve compression. Moreover, inhibition of TGF-β receptor signaling significantly reduced the expression of these fibrotic markers, highlighting the central role of the TGF-β/SMAD pathway in SSCT remodeling. Consistent with these observations, our meta-analysis identified elevated TGF-β levels in patients with CTS. Together, these findings suggest that, alongside inflammation, fibrotic pathways may represent an important mechanism underlying CTS and a potential target for future therapeutic strategies [30].

Chikenji et al. demonstrated significantly increased expression of TGF-β1, connective tissue growth factor (CTGF), and collagen III in the subsynovial connective tissue of patients with idiopathic CTS compared with controls. Furthermore, they reported a strong positive correlation between TGF-β1 and CTGF expression, suggesting activation of the TGF-β signaling pathway as a key mechanism underlying fibrotic tissue remodeling. These findings support the hypothesis that TGF-β-mediated fibrosis contributes to the pathogenesis of CTS. Consistent with these observations, our meta-analysis identified elevated TGF-β levels in patients with CTS, further reinforcing the potential role of pro-fibrotic pathways, in addition to inflammation, in the development and progression of the disease [31].

For TNF-α and IFN-α—despite their recognized roles in nerve injury mechanisms—no significant differences were found. This may indicate predominantly local expression or limitations in the sensitivity of the analytical methods used [11,13,17,19].

It is important to note that the local nature of the inflammatory response may partly explain the absence of significant serum changes for some cytokines. For example, Freeland et al. [22] reported elevated IL-6 and PGE2 levels only in tendon sheaths of patients, with no significant serum changes, highlighting the dominance of a local inflammatory environment in idiopathic CTS pathogenesis. These authors also suggested that an ischemia–reperfusion mechanism, rather than a classic inflammatory response, may be the predominant process leading to swelling, fibrosis, and secondary compression of the median nerve.

Some chemokines, such as CCL4 and CXCL10, may also be involved in neuropathic pain modulation through effects on sensory neuron excitability and macrophage recruitment to injured tissue. Immunohistochemical studies have shown that tendon sheath fibroblasts produce CCL- and CXCL-type chemokines, which may help maintain chronic, low-grade inflammation and promote fibrosis [21,32].

Chemokines (CCL5, CXCL8, CXCL10) and growth factors (VEGF, TGF-β1, BMP-7) are often elevated in CTS patients, especially those with comorbidities or more severe symptoms [11,12,17]. Serum levels of classical inflammatory cytokines (TNF-α, IL-1β, IL-6, IL-10) do not consistently differ between CTS patients and controls. Some markers (e.g., CRP, IL-6) may be higher in patients with more extensive symptoms, suggesting a link to mild systemic inflammation in certain CTS subgroups [11,19]. Although some chemokines and growth factors are elevated in CTS patients, classical inflammatory cytokines are not consistently increased [12,22]. This suggests the potential involvement of specific immune and fibrotic pathways, rather than a classic inflammatory response, in CTS pathogenesis, and that certain markers may help assess disease severity or the presence of comorbidities.

Recent evidence suggests that the pathogenesis of carpal tunnel syndrome extends beyond mechanical compression of the median nerve and involves a complex interplay between inflammation, oxidative stress, metabolic dysfunction, and tissue remodeling. In particular, diabetes mellitus and insulin resistance may amplify inflammatory and fibrotic signaling through chronic low-grade inflammation, endothelial dysfunction, oxidative stress, and impaired nerve regeneration, thereby increasing the susceptibility of the median nerve to compression injury. These findings support a multifactorial model of CTS pathogenesis rather than a purely mechanical etiology. Furthermore, in addition to cytokines and chemokines evaluated in the present meta-analysis, previous studies have reported associations between CTS and other inflammatory biomarkers, including C-reactive protein (CRP), matrix metalloproteinases (MMPs), adhesion molecules, and markers of oxidative stress. Although these biomarkers were not quantitatively analyzed due to insufficient and heterogeneous data, they further support the concept that inflammatory and metabolic pathways contribute to CTS development and progression [33].

### 4.4. Strengths, Limitations, and Clinical Implications

This is the first meta-analysis to comprehensively examine associations between a broad spectrum of inflammatory markers—including chemokines, interleukins, and growth factors—and CTS.

Importantly, the observational design of all included studies precludes any inference of causality. The elevated levels of inflammatory markers observed in CTS patients may represent a consequence of nerve compression, a contributing factor, or an epiphenomenon related to comorbidities or systemic inflammation. Therefore, causal relationships cannot be established based on the current evidence.

The inclusion of only studies with a control group and measurement of markers prior to treatment reduces potential confounding factors. However, several limitations should be acknowledged. The number of available studies is limited, with varying size and quality. For some cytokines, data came only from two studies, limiting generalizability. Additionally, the lack of correlation between marker levels and clinical severity or electrophysiological findings represents a significant research gap.

The absence of differences in IL-6 levels—one of the most widely reported cytokines in CTS literature—underscores the inconsistency of available data and the need for further well-designed studies. The identification of elevated CCL2, CCL5, IL-4, VEGF, and TGF-β levels in CTS suggests their potential utility as diagnostic tools or therapeutic targets. Their roles in fibrosis, nerve injury, and microcirculatory disturbances support the concept of a complex, multifactorial CTS pathophysiology extending beyond the classic mechanical compression theory. Future research should focus on prospective analyses correlating biomarker levels with clinical symptoms, treatment outcomes, and disease progression.

The potential influence of comorbidities on inflammatory biomarker levels should be considered when interpreting the findings of this meta-analysis. Although most included studies excluded participants with conditions known to alter cytokine and chemokine profiles, such as diabetes mellitus, obesity, metabolic syndrome, hypothyroidism, and inflammatory diseases, complete elimination of their potential impact cannot be ensured. Differences in exclusion criteria, the presence of unrecognized or subclinical conditions, and inconsistent reporting of comorbidities across studies may have contributed to the observed heterogeneity. Consequently, residual confounding cannot be ruled out, and the findings should be interpreted with appropriate caution.

Another important limitation is the variability in laboratory methods used to quantify cytokines and chemokines across the included studies. Differences in analytical platforms, including enzyme-linked immunosorbent assays (ELISAs) and multiplex immunoassays, as well as variations in assay sensitivity, specificity, calibration, and sample processing protocols, may have affected the reported biomarker concentrations and contributed to inter-study heterogeneity. Consequently, the pooled effect estimates should be interpreted cautiously. Future studies should adopt standardized analytical protocols to improve the comparability, reproducibility, and reliability of biomarker measurements.

Finally, the relatively small number of studies included in the quantitative synthesis represents an additional limitation. As fewer than ten studies were available for each meta-analysis, the statistical power of Egger’s test to detect publication bias was limited. Therefore, the absence of statistically significant publication bias should not be interpreted as definitive evidence of its absence, as the test may have been underpowered to detect small-study effects.

### 4.5. Implications for Current Practise and Future Research

The findings of this meta-analysis highlight that, beyond mechanical compression, carpal tunnel syndrome (CTS) may involve a relevant inflammatory and potentially pro-fibrotic component. Elevated levels of selected mediators—such as CCL2, CCL5, CXCL10, CXCL8, IL-4, VEGF, and TGF-β—suggest that immune and tissue-remodeling pathways may contribute to disease pathophysiology. However, these observations should be interpreted cautiously, as the current evidence does not provide longitudinal data or diagnostic accuracy measures sufficient to support the routine use of serum biomarkers for diagnosis or disease monitoring.

At present, clinical assessment and electrodiagnostic testing remain the cornerstone of CTS diagnosis. While inflammatory markers may offer additional pathophysiological insight and could be of interest in research settings or in the stratification of patient subgroups, their role in clinical decision-making is not yet established. Furthermore, the identification of pro-fibrotic and angiogenic factors such as TGF-β and VEGF may indicate potential therapeutic targets, although this requires further validation in well-designed prospective studies.

Overall, integrating the immunological profile into routine clinical evaluation of CTS can contribute to the understanding of disease mechanisms and support the development of more personalized therapeutic approaches in the future.

## 5. Conclusions

- Elevated levels of inflammatory markers, including CCL2, CCL5, CXCL10, CXCL8, IL-4, VEGF, and TGF-β, were observed in patients with CTS compared with controls, indicating an association between these cytokines/chemokines and the presence of CTS.
- The available observational evidence suggests that CTS is associated with increased levels of selected cytokines and chemokines involved in inflammatory pathways. However, these findings should not be interpreted as evidence of causality and have, at the present time, a limited clinical use. Further prospective studies are required to determine their clinical relevance and potential use for therapeutic purposes.

## Figures and Tables

**Table 1:** Study characteristics.

Study (Country)	Study Description							Sample Description		Affected Side		
	Observational	NOS				n Randomized/Analyzed	Study Focus	Age (Mean SD)	Male (%)	Left (n)	Right (n)	Bilateral (n)
		Selection	Comparability	Exposure	Conclusion							
Ajeena et al., 2021 [20] (Iraq)	Cross-sectional	**	0	**	4/Low	64/64	Exploring associations between RANTES and CTS	44.9 (7.8)	7 (10.94)	13	26	25
Baričić et al., 2023 [11] (Croatia)	Case–control	****	*	***	8/High	75/31	Exploring associations between cytokines and CTS	65.12 (5.5)	29 (38.67)	nd	nd	nd
Fattah et al., 2023 [21] (Egypt)	Case–control	****	*	***	8/High	200/100	Exploring associations between chemical mediators in serum and CTS	46.95 (12.9)	70 (35)	26	25	49
Freeland et al., 2002 [22] (USA)	Case–control	****	*	**	7/Good	62/41	Serum inflammatory cytokine levels in CTS patients compared to healthy individuals	46.70 (nd)	10 (16.13)	nd	nd	nd
Karimi et al., 2021 [19] (Iran)	Case–control	***	*	**	6/Moderate	80/40	Exploring associations between cytokines and chemokines and CTS	45.20 (8.47)	0 (0)	10	14	16
Moalem-Taylor et al., 2017 [17] (Australia)	Case–control	****	**	**	8/High	52/26	Exploring associations between inflammatory markers and CTS	55.20 (10.30)	13 (25)	2	7	17
Sandy-Hindmarch et al., 2022 [13] (United Kingdom)	Case–control	****	**	***	9/High	76/55	Exploring associations between inflammatory markers and CTS	63.50 (nd)	25 (32.89)	nd	nd	nd

NOS—Newcastle–Ottawa Scale, nd = not determined, * Score according to the Newcastle–Ottawa Scale (NOS) (One * represents one score.).

## Data Availability

The data are available upon reasonable request from the corresponding author.

## Supplementary Materials

The following supporting information can be downloaded at: https://www.mdpi.com/article/10.3390/jcm15145545/s1, Table S1: PRISMA 2020 for Abstracts Checklist [34]; Table S2: PRISMA 2020 Checklist [34].

## Abbreviations

CCL2	Chemokine ligand 2
CCL4	Chemokine ligand 4
CCL5	Chemokine ligand 5
CTS	Carpal tunnel syndrome
CXCL8	Chemokine ligand 8
CXCL10	Chemokine ligand 10
IFN-α	Interferon alpha
IL-1β	Interleukin 1 beta
IL-4	Interleukin 4
IL-6	Interleukin 6
IL-9	Interleukin 9
IL-10	Interleukin 10
ROS	Reactive oxygen species
SDM	Standardized mean difference
TGF-β	Transforming Growth Factor beta
TNF-α	Tumor Necrosis Factor alpha
VEGF	Vascular Endothelial Growth Factor
